# Investigation of Solitary wave solutions for Vakhnenko-Parkes equation via exp-function and *Exp*(−*ϕ*(*ξ*))-expansion method

**DOI:** 10.1186/2193-1801-3-692

**Published:** 2014-11-25

**Authors:** Harun-Or Roshid, Md Rashed Kabir, Rajandra Chadra Bhowmik, Bimal Kumar Datta

**Affiliations:** Department of Mathematics, Pabna University of Science and Technology, Dhaka - Pabna Hwy, Pabna, 6600 Bangladesh

## Abstract

In this paper, we have described two dreadfully important methods to solve nonlinear partial differential equations which are known as exp-function and the exp(−*ϕ*(*ξ*)) -expansion method. Recently, there are several methods to use for finding analytical solutions of the nonlinear partial differential equations. The methods are diverse and useful for solving the nonlinear evolution equations. With the help of these methods, we are investigated the exact travelling wave solutions of the Vakhnenko- Parkes equation. The obtaining soliton solutions of this equation are described many physical phenomena for weakly nonlinear surface and internal waves in a rotating ocean. Further, three-dimensional plots of the solutions such as solitons, singular solitons, bell type solitary wave i.e. non-topological solitons solutions and periodic solutions are also given to visualize the dynamics of the equation.

## 1. Introduction

The effort in finding exact solutions to nonlinear equations is witnessed significant curiosity and progress in finding solutions to nonlinear partial differential equations (NPDEs) that resemble physical phenomena. The nonlinear wave phenomena observed in fluid dynamics, plasma and optical fibers are often modeled by the bell (i.e. non-topological solitons) shaped sech solutions and the kink (i.e. topological solitons) shaped tanh solutions. Both mathematicians and physicists have devoted considerable effort of research regarding this matter. A peek at the literature reveals a lot of effective methods that solve this type of NPDEs.

For instance the inverse scattering transform (Ablowitz and Clarkson 1991; Vakhnenko and Parkes 2002; Vakhnenko and Parkes 2012a; Vakhnenko and Parkes 2002b), the complex hyperbolic function method (Zayed et al. 2006; Chow 1995), the rank analysis method (Feng 2000), the ansatz method (Hu 2001a; Hu 2001b; Majid et al. 2012), the (*G*′/*G*) -expansion method (Wang et al. 2008; Roshid et al. 2013a; Bekir 2008; Roshid et al. 2013b; Zhang 2008; Alam 2013), the modified simple equation method (Jawad et al. 2010), the exp-functions method (He and Wu 2006), the Hirota method (Hirota 1971), the sine-cosine method (Wazwaz 2004), the tanh-function method (Parkes and Duffy 1996), extended tanh-function method (Fan 2000; Parkes 2010a; Parkes 2010b), the Jacobi elliptic function expansion method (Liu 2005; Chen and Wang 2005), the F-expansion method (Wang and Zhou 2003; Wang and Li 2005), the Backlund transformation method (Miura 1978), the Darboux transformation method (Matveev and Salle 1991), the homogeneous balance method (Wang 1995; Zayed et al. 2004; Wang 1996), the Adomian decomposition method (Adomain 1994; Wazwaz 2002), the auxiliary equation method (Sirendaoreji and Sun 2003; Sirendaoreji 2007), the exp(−*ϕ*(*ξ*)) -expansion method (Khan and Akbar 2013) and so on.

Recently, a remarkable and important discover has been made by Vakhnenko and Parkes (Vakhnenko and Parkes 1998), who have confirmed an integrable equation as follows:
1

The traveling wave solutions of this Vakhnenko-Parkes equation was investigated in (Kangalgil and Ayaz 2008; Parkes 2010b; Gandarias and Bruzon 2009; Yasar 2010; Abazari 2010; Liu and He 2013, Ostrovsky 1978) and Liu (Liu and He 2013) found traveling wave solutions of this equations by improved (*G*′/*G*) -expansion method with auxiliary equation *GG*″ = *AG*^2^ + *BGG*′ + *C*(*G*′)^2^.

In this paper, we investigate the traveling wave solutions of the Vakhnenko-Parkes equation (1) via two methods namely the Exp-function and the exp(−*ϕ*(*ξ*)) -expansion methods.

The rest of the paper is organized as follows: In section 2, we build up an introduction of exp-function and the exp(−*ϕ*(*ξ*)) -expansion method. By these methods, we gain the exact solutions of Vakhnenko-Parkes equation in section 3. In section 4, we out line results and discussion of the achieved solutions. Finally, some conclusions are drawn in the section 5.

## 2. The methodologies

In this section, we will go over the main points of the exp-function method and the exp(−*ϕ*(*ξ*)) -expansion method to raise the rational solitary wave and periodic wave solutions for the Vakhnenko-Parkes equation which have been paid attention by many researchers in mathematical physics.

Consider a nonlinear equation with two independent variable *x* and *t*, is given by
2

where *U* = *U*(*x,t*) is an unknown function, *P* is a polynomial in *U* = *U*(*x,t*) and its partial derivatives, in which the highest order derivatives term and nonlinear terms are involved.

Combining the independent variable *x* and *t* into one traveling wave variable *ξ* = *x* ± *wt*, we suppose that
3

The travelling wave variable (3) permits us to convert the Eq. (2) to an ODE for *u* = *u*(*ξ*) is
4

### 2.1. The exp-function method

We now discuss the exp-function method to solve partial differential equation Eq. (1).

**Step-2.1.1.** Assume the solution of the Eq. (1) can be expressed in the following form (He and Wu, 2006):
5

where *c*, *d*, *p* and *q* are positive unknown integers that could be determine subsequently, *a*_*n*_ and *b*_*m*_ are unknown constants, Eq. (5) can be re-written in the following form:
6

**Step-2.1.2:** To determine the values of *c* and *p*, we balance the highest order linear term with the highest order nonlinear term in Equation Eq. (4). Similarly, to determine the values of *d* and *q*, we have to balance the lowest order linear term with the lowest order nonlinear term in Equation Eq. (4). This confirms the determination of the values of *c*, *d*, *p* and *q*.

**Step-2.1.3:** Inserting the values of *c*, *d*, *p* and *q* into Eq. (6) and then substituting Eq. (6) into Eq. (4) and simplifying, we attain;
7

Then collecting all coefficient *Cj* and setting each of them to zero, yields a system of algebraic equations for *a*_*c*_’s and *b*_*p*_’s. Then unknown *a*_*c*_’s and *b*_*p*_’s can be evaluated by solving the system of algebraic equations with the help of maple-13. Substituting these values into Eq. (6), we gain traveling wave solutions of the Eq. (1).

### 2.2. The exp(−*ϕ*(*ξ*)) -expansion method

**Step 2.2.1.** Assume that the solution of ODE (4) can be expressed by a polynomial in exp(−*ϕ*(*ξ*)) as follows:
8

where *ϕ*′(*ξ*) satisfies the ODE
9

The well-known solutions of the ODE (9) are as follows:
1011121314

*l*_*i*_, *w*, *λ*; *i* = 0, ⋯ ⋯, *m* and *μ* are constants to be determined later, *l*_*m*_ ≠ 0, the positive integer *m* can be determined by considering the homogeneous balance between the highest order derivatives and nonlinear terms arising in the ODE(4).

**Step 2.2.2**. By substituting Eq. (8) into Eq. (4) and using the ODE (9), and then collecting all terms with the same order of exp(−*ϕ*(*ξ*)) together, the left hand side of Eq. (4) is converted into new polynomial in exp(−*ϕ*(*ξ*)). Setting each coefficient of this polynomial to zero, yields a system of algebraic equations for *l*_*i*_, ⋯ *w*, *λ*; *i* = 0, ⋯ ⋯, *m* and *μ*. Solving the system of algebraic equations and substituting *l*_*i*_, ⋯ *w*; *i* = 0, ⋯ ⋯, *m*, and the general solutions of Eq. (9) into Eq. (8). We have more traveling wave solutions of nonlinear evolution equation Eq. (1).

## 3. Application

In this section, we exert the exp-function method and the exp(−*ϕ*(*ξ*)) -expansion method to construct the rational solitary wave, non-topological soliton, periodic wave solutions for some nonlinear evolution equations in mathematical physics via the Vakhnenko-Parkes equation Eq. (1).

Inserting Eq. (3) into Eq. (1), we amend the Eq. (1) into the ODE:
15

Integrating Eq. (15) with respect to *ξ* and setting the integration constant equal to zero yields
16

### 3.1. Solution of Vakhnenko- Parkes equation via the exp-function method

Now, we apply the Exp-function method to create the generalized traveling wave solutions of the Vakhnenko- Parkes Eq. (1).

According to Step 2.1.1 in the Exp-function method, the solution of Eq. (16) can be written in the form of Eq. (6). To determine the values of *c* and *p*, according to Step 2.1.2, we balance the term of the highest order in *uu*″ and the highest nonlinear terms *u*^3^ in Eq. (16). With the aid of computational software Maple 13, yields *p* = *c*. To find out the values of *q* and *d*, we balance the term of lowest order *uu*″ in Eq. (16) with lowest order nonlinear term *u*^3^, with the aid of computational software Maple 13, yields to result *q* = *d.* The parameters are free, so we can arbitrarily prefer the values of c and d, but the ultimate solution does not depend upon the choices of them.

**Case 1:** Suppose *p* = *c* = 1 and *q* = *d* = 1.
17

Since there are some free variables, for simplicity, we presume *b*_1_ = 1.
18

Now, substituting Eq. (18) into Eq. (16) and by employing the computer algebra, such as Maple 13, we gain


Where *A* = (e^2*ξ*^ + b_0_e^*ξ*^ + b_‒ 1_)^4^,


Setting these equations to zero and solving the system of algebraic equations with the aid of commercial software Maple-13, we achieve the following solution.


Setting these values in the Eq. (18) we acquire the solution
19

If we set

If we choose
20

**Case 2:** Suppose *p* = *c* = 2 and *q* = *d* = 1.
21

Since there are some free parameters, for simplicity, we imagine *b*_2_ = 1, *b*_− 1_ = 0.
22

Executing the same procedure as described in case-1, we gain


Setting these values in the Eq. (22) we acquire the solution
23

which is same obtain in the previous case-1.

**Case 3:** Suppose *p* = *c* = 2 and *q* = *d* = 2.
24

Since there are some free parameters, for simplicity, we presume *a*_− 2_ = *a*_− 1_ = 0, *b*_− 2_ = *b*_− 1_ = 0, *b*_1_ = 1_._25

Executing the same procedure as described in the case-1 and in the case-2, we attain


Hence require solution is
26

where *ξ* = *x* − *wt*.

This is also similar solutions achieved in the previous cases and so we should not repeat the procedure again and again for different values of the parameters. Actually the solution is a bell shape soliton solution which referred to as non-topological solitons solution. But in generally, we can obtain all of the above solutions and another family of solutions in case 4.

**Case 4:** Suppose *p* = *c* = 1 and *q* = *d* = 1.
27

Now, substituting Eq. (27) into Eq. (15) and by employing the computer algebra, such as Maple 13, we gain


Where *A* = (*b*_1_e^2*ξ*^ + b_0_e^*ξ*^ + b_‒ 1_)^5^, others are omitted for simplicity and setting these equations to zero and solving the system of algebraic equations with the aid of commercial software Maple-13, we achieve the following solution.(i) (ii) 

The solution (i) is same obtained in case 1.

Setting these values of (ii) in the Eq. (18) we acquire the solution
28

If we choose
29

Or if choose
30

**Remark-1:** We have the solution (19) in the form via Exp-function method, 

It note that if *b*_0_ > 0 and exp(−*x*_0_) = 2/*b*_0_ then it can be written  and if *b*_0_ <0 and exp(−*x*_0_) = 2/|*b*_0_| then it can be written . These two solutions are just solutions *u*_11_ and *u*_12_ in Parkes (Parkes 2010b) with *k* = 1/2.

And for the solution (29) in the form via Exp-function method, 

It note that if *b*_1_/*b*_0_ > 0 and exp(−*x*_0_) = 2*b*_1_/*b*_0_ then it can be written  and if *b*_1_/*b*_0_ < 0 and exp(−*x*_0_) = 2*b*_1_/|*b*_0_| then it can be written . These two solutions are just solutions *u*_21_ and *u*_22_ in Parkes (Parkes 2010b) with *k* = 1/2.

### 3.2. Solutions of Vakhnenko- Parkes equation via the exp(−*ϕ*(*ξ*)) -expansion method

Balance the highest order derivate term *uu*″ with the highest nonlinear terms *u*^3^ in Eq. (16), we obtain *m* = 2, so assume the equation Eq. (1) has the solution
31

Inserting Eq. (31) into Eq. (16) and using the ODE (9), and then collecting all terms with the same order of exp(−*ϕ*(*ξ*)) together, Eq. (16) is converted into new polynomial in exp(−*ϕ*(*ξ*)). Setting each coefficients of this polynomial is to zero, yields a system of algebraic equations for *l*_0_, *l*_1_, *l*_2_ , *λ*; and *μ* which are as follows:


Solving the system of algebraic equations and we obtained *l*_0_ = − 6*μ*, *l*_1_ = − 6*λ*, *l*_2_ = − 6. Substituting the values of *l*_0_, *l*_1_, *l*_2_ in the general solutions of Eq. (9) achieve more traveling wave solutions of nonlinear evolution equation Eq. (1) as follows:

When *λ*^2^ − 4*μ* > 0, *μ* ≠ 0, then
32

When *λ*^2^ − 4*μ* < 0, then
33

When *λ*^2^ − 4*μ* > 0, *μ* = 0, then
34

When *λ*^2^ − 4*μ* = 0, *μ* ≠ 0, *λ* ≠ 0, then
3536

**Remark-2:** All of the solutions presented in this latter have been checked with Maple by putting them back into the original equations.

## 4. Results and discussion

In this paper we exerted the exp-function methods and the exp(−*ϕ*(*ξ*)) -expansion method as useful mathematical tools to construct topological soliton, non-topological soliton, periodic wave solutions for the Vakhnenko- Parkes equation. The methods have successfully handled with the aid of commercial software Maple-13 that greatly reduces the volume of computation and improves the results of the equation. We have achieved a family of solutions via exp-function method. It is worth declaring that some of our obtained solutions via the exp(−*ϕ*(*ξ*)) -expansion method is in good agreement with already published results which is presented in the Tables 1 and 2. The others are completely new solutions achieved by exp(−*ϕ*(*ξ*)) -expansion method.

**Table 1:** **Comparison between Liu and He’s (Liu and He** 2013 **) solutions and our solutions**

Liu and He (Liu and He 2013 )	Our solution
(i) If *A* = 1, *B* = 0, *C* − 1 = *μ* and *c*_2_ = 0 then from equation (19) we obtain	(i) If *λ* = 0, *C* = 0 then our solutions (33) reduced to
(ii) If *A* = 1, *B* = 0, *C* − 1 = *μ* and *c*_2_ = 0 then from equation (20) we obtain	(ii) If *λ* = 0, *C* = 0 then our solutions (32) reduced to

**Table 2:** **Comparison between Parkes’s (Parkes** 2010b **) solutions and our solutions**

Parkes’s (Parkes 2010b )	Our solution
(i) If *k* ^2^ = *μ*, *η* = *ξ* + *C* then solution *u*_14_ we obtain	(i) If *λ* = 0, then our solutions (33) reduced to
(ii) If *k* ^2^ = − *μ*, *η* = *ξ* + *C* then from solution *u*_12_ we obtain	(ii) If *λ* = 0, then our solutions (28) reduced to
(iii) If *k* = *λ*/2, *η* = *ξ* + *C* then solution *u*_12_ we obtain	(iii) Eq. (34) can be simplified to gives
(iv) If *η* = *ξ* + *C* + *λ*/2 then solution *u*_3_ we obtain	(iv) Eq. (35) can be simplified to gives

### 4.1. Physical interpretation

**Figure 1:**
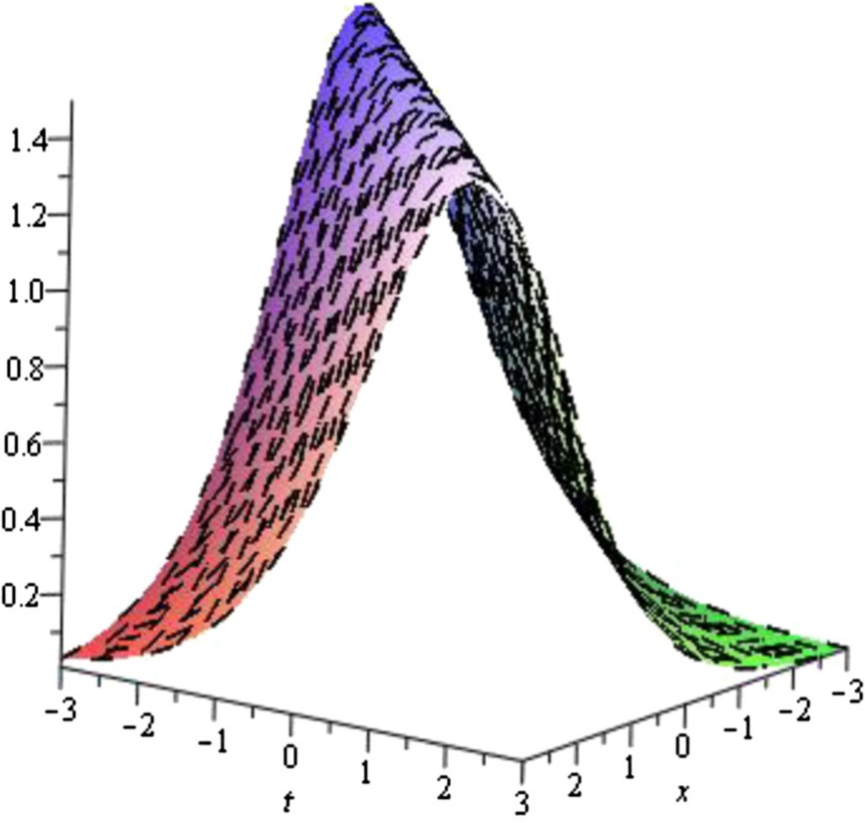
**Bell shape (non-topological) soliton solution of the Eq.** (19 ) **for the parameters** ***b***_**0**_ **= 4,** ***w*** **= 1.**

**Figure 2:**
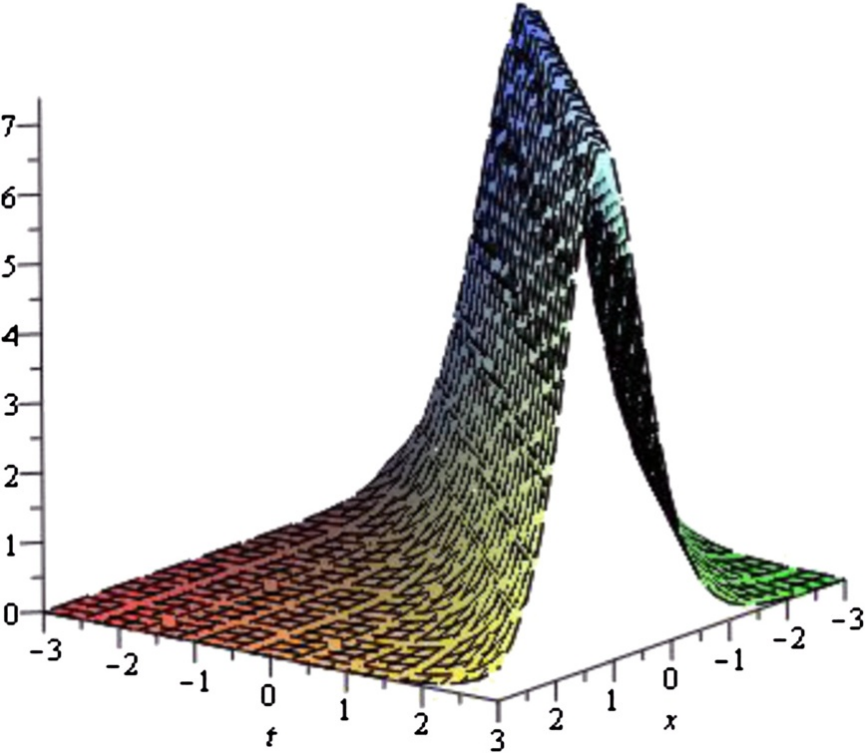
**Cuspon soliton solution of the Eq.** (32 ) **for the parameters** ***λ*** **= 3,** ***μ*** **=** ***c*** **=** ***w*** **= 1** **.**

In this subsection, we describe the physical interpretation of the solutions for the Vakhnenko- Parkes equation. Solitons are solitary waves with stretchy dispersion possessions, which described many physical phenomena in soliton physics. Soliton preserve their shapes and speed after colliding with each other. Soliton solutions also give ascend to particle-like structures, such as magnetic monopoles etc. The solution (19) in Figure 1 of the equation (1) is represented the exact Bell type solitary (non-topological soliton) wave solution for the parameters *b*_0_ = 4, *w* = 1 with − 3 ≤ *x*, *t* ≤ 3 via exp-function method. Since second family Eq. (30) has a constant different with first family it figure is also the exact Bell type solitary (non-topological soliton) wave solution. Others solutions via exp-function method are similar to this solution or can be obtained from this solution which profiles are similar to the Figure 1. The solution (32) obtained by the exp(−*ϕ*(*ξ*)) -expansion method is cuspon whose shape is depicted in the Figure 2 for the parameters *λ* = 3, *μ* = *c* = *w* = 1 with − 3 ≤ *x*, *t* ≤ 3.

**Figure 3:**
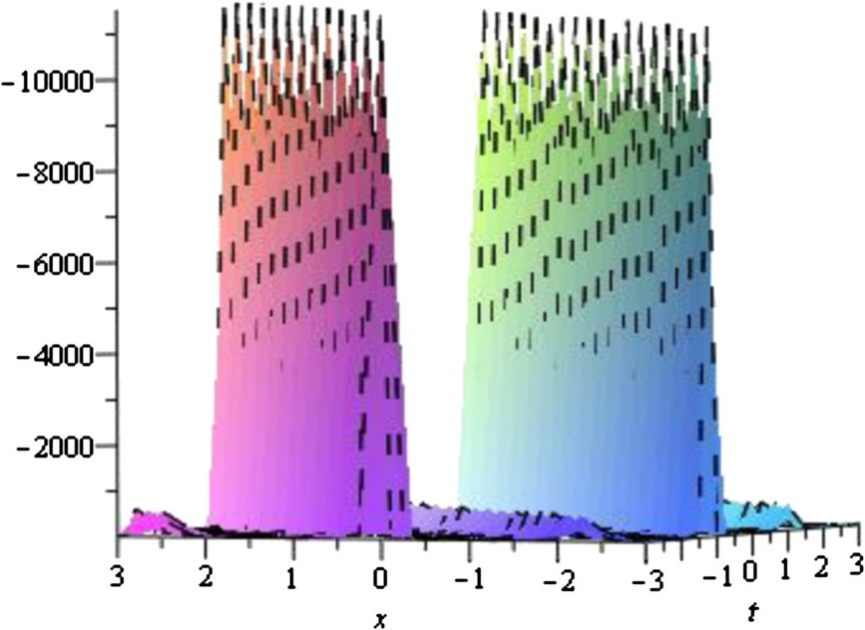
**Periodic solution of the Eq.** (33) **for the parameters** ***λ*** **= 1**, ***μ*** = ***c*** **= 2**, ***w*** **= 1** **.**

The solution (33) of the equation Eq. (11) is presented the periodic travelling wave solution for various values of the physical parameters. The Figure 3 has been shown the shape of the solution (33) for the parameters *λ* = 1, *μ* = *c* = 2, *w* = 1 with − 3 ≤ *x*, *t* ≤ 3.

**Figure 4:**
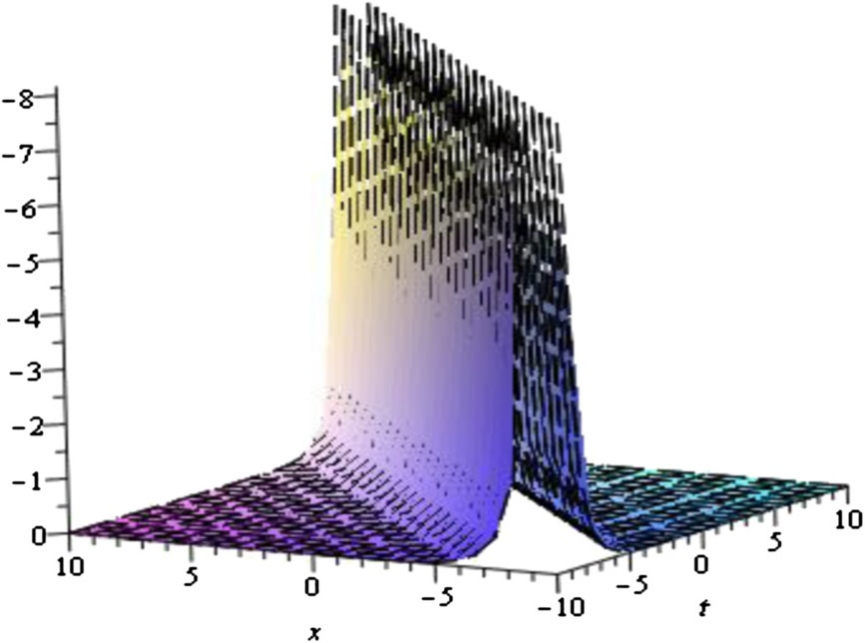
**Singular soliton solution of the Eq.** (34) **for the parameters** ***λ*** **=** ***w*** **= 1,** ***μ*** **=** ***c*** **= 0** **.**

Solutions (34) of the equation Eq. (1) represent singular soliton solution for the parameters *λ* = *w* = 1, *μ* = *c* = 0 with − 3 ≤ *x*, *t* ≤ 3 whose shape is given by the Figure 4.

**Figure 5:**
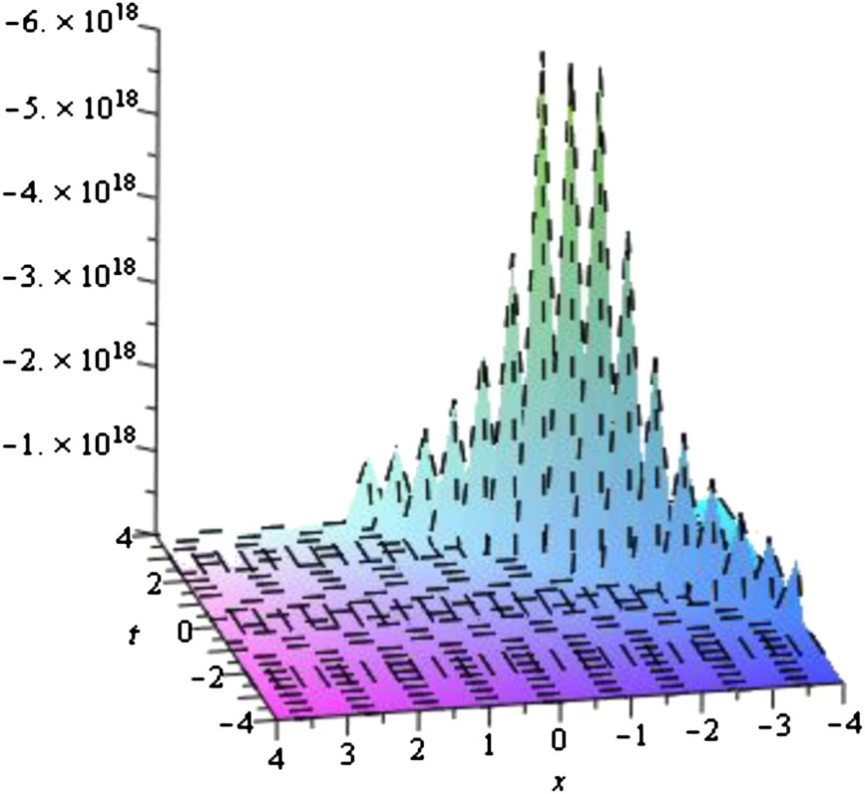
**Multiple soliton solution of the Eq.** (35) **for the parameters** ***λ*** **=** ***c*** **= 2,** ***μ*** **=** ***c*** **= 1** **.**

Finally, solution (35) and (36) are similar type solutions and they represent the multiple soliton solution. Omitting one figure we depicted the Figure 5 of the Eq. (35) for the parameters *λ* = *w* = 1, *μ* = *c* = 0 with − 3 ≤ *x*, *t* ≤ 3.

### 4.2. Graphical representations

The graphical illustrations of the solutions are given below in the figures (Figures 1, 2, 3, 4 and 5) with the aid commercial software of Maple-13.

## 5. Conclusion

In this research some new solitary wave solutions of the Vakhnenko-Parkes equation is found using the exp-function method and the exp(−*ϕ*(*ξ*)) -expansion method. As a results two family of bell type solitary wave solutions Eq. (19) or Eq. (26) and Eq. (30) using exp-function method and five solutions Eq. (32)-Eq. (36) including cuspon, singular soliton, multiple soliton and periodic solutions are achieved via exp(−*ϕ*(*ξ*)) -expansion method of the Vakhnenko- Parkes equation exist for real sense depends on different relevant physical parameters. Numerical results of the solutions for real sense by using Maple software have been shown graphically and discussed. This will have a good sense to encourage the extensive application of the equations.

## References

[CR1] Abazari R (2010). Application of (*G’/G*)-expansion method to traveling wave solutions of three nonlinear evolution equation. Comput Fluids.

[CR2] Ablowitz MJ, Clarkson PA (1991). Soliton, Nonlinear Evolution Equations and Inverse Scattering.

[CR3] Adomain G (1994). Solving Frontier Problems of Physics: The Decomposition Method.

[CR4] Alam MN, Akbar MA, Roshid HO (2013). Study of nonlinear evolution equations to construct traveling wave solutions via the new approach of generalized (*G’/G*)-expansion method. Math Stat.

[CR5] Bekir A (2008). Application of the (*G’/G*)-expansion method for nonlinear evolution equations. Phys Lett A.

[CR6] Chen Y, Wang Q (2005). Extended Jacobi elliptic function rational expansion method and abundant families of Jacobi elliptic functions solutions to (1 + 1)-dimensional dispersive long wave equation. Chaos, Solitons Fractals.

[CR7] Chow KW (1995). A class of exact periodic solutions of nonlinear envelope equation. J Math Phys.

[CR8] Fan E (2000). Extended tanh-function method and its applications to nonlinear equations. Phys Lett A.

[CR9] Feng X (2000). Exploratory approach to explicit solution of nonlinear evolutions equations. Int J Theo Phys.

[CR10] Gandarias ML, Bruzon MS (2009). Symmmetry relations and exact solutions for the Vakhnenko equation. The XXI congreso de ecuaciones diferenciales y aplicaciones, XI congreso de matematica aplicada, ciudad real, 21-25 Septiembre, 16-22.

[CR11] He JH, Wu XH (2006). Exp-function method for nonlinear wave equations. Chaos Solitons Fractal.

[CR12] Hirota R (1971). Exact solution of the KdV equation for multiple collisions of solutions. Phys Rev Lett.

[CR13] Hu JL (2001). Explicit solutions to three nonlinear physical models. Phys Lett A.

[CR14] Hu JL (2001). A new method for finding exact traveling wave solutions to nonlinear partial differential equations. Phys Lett A.

[CR15] Jawad AJM, Petkovic MD, Biswas A (2010). Modified simple equation method for nonlinear evolution equations. Appl Math Comput.

[CR16] Kangalgil F, Ayaz F (2008). New exact traveling wave solutions for the Ostrovsky equation. Phys Lett A.

[CR17] Khan K, Akbar MA (2013). Application of exp(−Φ(*η*))-expansion method to find the exact solutions of modified Benjamin-Bona-Mahony equation. World Appl Sci J.

[CR18] Liu D (2005). Jacobi elliptic function solutions for two variant Boussinesq equations. Chaos, Solitons Fractals.

[CR19] Liu XH, He C (2013). New traveling wave solutions to the Vakhnenko-Parks equation. ISRN Math Phys.

[CR20] Majid F, Triki H, Hayat T, Aldossary OM, Biswas A (2012). Solitary wave solutions of the Vakhnenko-Parkes equation. Nonlinear Anal.

[CR21] Matveev VB, Salle MA (1991). Darboux Transformation and Solitons.

[CR22] Miura MR (1978). Backlund Transformation.

[CR23] Ostrovsky LA (1978). Nonlinear internal waves in a rotating ocean. Oceanology.

[CR24] Parkes EJ (2010). Observations on the basic (*G’/G*)-expansion method for finding solutions to nonlinear evolution equations. Appl Math Comput.

[CR25] Parkes EJ (2010). A note on “New travelling wave solutions to the Ostrovsky equation”. Appl Math Comput.

[CR26] Parkes EJ, Duffy BR (1996). An automated tanh-function method for finding solitary wave solutions to nonlinear evolution equations. Comput Phys Commun.

[CR27] Roshid HO, Rahman N, Akbar MA (2013). Traveling waves solutions of nonlinear Klein Gordon equation by extended (G′/G)-expasion method. Ann Pure Appl Math.

[CR28] Roshid HO, Alam MN, Hoque MF, Akbar MA (2013). A new extended (*G’/G*)-expansion method to find exact traveling wave solutions of nonlinear evolution equations. Math Stat.

[CR29] Sirendaoreji (2007). Auxiliary equation method and new solutions of Klein-Gordon equations. Chaos Solitions Fractals.

[CR30] Sirendaoreji, Sun J (2003). Auxiliary equation method for solving nonlinear partial differential equations. Phys Lett A.

[CR31] Vakhnenko VO, Parkes EJ (1998). The two loop soliton solution of the Vakhnenko-equation. Nonlinearity.

[CR32] Vakhnenko VO, Parkes EJ (2002). The calculations of multi-soliton solutions of the Vakhnenko equation by the inverse scattering method. Chaos, Solitons Fractals.

[CR33] Vakhnenko VO, Parkes EJ (2012). Solutions associated with discrete and continuous spectrums in the inverse scattering method for the Vakhnenko-Parkes equation. Progr Theor Phys.

[CR34] Vakhnenko VO, Parkes EJ (2012). The singular solutions of a nonlinear evolution equation taking continuous part of the spectral data into account in inverse scattering method. Chaos, Solitons Fractals.

[CR35] Wang M (1995). Solitary wave solutions for variant Boussinesq equations. Phy Lett A.

[CR36] Wang ML (1996). Exact solutions for a compound KdV-Burgers equation. Phys Lett A.

[CR37] Wang ML, Li XZ (2005). Extended F-expansion method and periodic wave solutions for the generalized Zakharov equations. Phys Lett A.

[CR38] Wang ML, Zhou YB (2003). The periodic wave solutions for the Klein-Gordon-Schrodinger equations. Phys Lett A.

[CR39] Wang ML, Li XZ, Zhang J (2008). The (*G’/G*)-expansion method and traveling wave solutions of nonlinear evolution equations in mathematical physics. Phys Lett A.

[CR40] Wazwaz AM (2002). Partial Differential Equations: Method and Applications.

[CR41] Wazwaz AM (2004). A sine-cosine method for handle nonlinear wave equations. Appl Math Comput Model.

[CR42] Yasar E (2010). New traveling wave solutions to the Ostrovsky equation. Appl Math and Comput.

[CR43] Zayed EME, Zedan HA, Gepreel KA (2004). On the solitary wave solutions for nonlinear Hirota-Sasuma coupled KDV equations. Chaos Solitons Fractals.

[CR44] Zayed EME, Abourabia AM, Gepreel KA, Horbaty MM (2006). On the rational solitary wave solutions for the nonlinear Hirota CSatsuma coupled KdV system. Appl Anal.

[CR45] Zhang S, Tong J, Wang W (2008). A generalized (*G’/G*)-expansion method for the mKdV equation with variable coefficients. Phys Lett A.

